# Crystal Plasticity Modeling of Anisotropic Hardening and Texture Due to Dislocation Transmutation in Twinning

**DOI:** 10.3390/ma11101855

**Published:** 2018-09-28

**Authors:** Robert M. Allen, Laszlo S. Toth, Andrew L. Oppedal, Haitham El Kadiri

**Affiliations:** 1 Department of Mechanical Engineering, Mississippi State University, Mississippi State, MS 39762, USA; rma140@msstate.edu (R.M.A.); aoppedal@cavs.msstate.edu (A.L.O.); 2 Center for Advanced Vehicular Systems, Mississippi State University, Mississippi State, MS 39762, USA; 3 Laboratory of Excellence on Design of Alloy Metals for Low-Mass Structures (DAMAS), Université de Lorraine, 57045 Metz, France; laszlo.toth@univ-lorraine.fr; 4 Laboratoire d’Etude des Microstructures et de Mécanique des Matériaux (LEM3), CNRS UMR 7239, Université de Lorraine, 57045 Metz, France

**Keywords:** magnesium, twinning, hardening, dislocations, crystal plasticity, self consistent methods, modeling, simulation

## Abstract

In crystalline materials, dislocations are three-dimensional lattice distortions that systematically distort twin interfaces that they encounter. This results in dislocation dissociation events and changes in the atomic structure of the interface. The manner in which the interface distorts drive the product of the dissociation event, and consequently, the incident dislocation core and the magnitude and relative direction of the Burgers vector govern these slip-twin interaction phenomena. Recent characterization studies using transmission electron microscopy as well as advanced molecular dynamic simulations have shown that slip dislocations, whether striking or struck by a {10$\bar{1}$2} twin boundary, dissociate into a combination of twinning disconnections, interfacial disclinations (facets), jogs, and other types of dislocations engulfed inside the twin domains, called transmuted dislocations. While twinning disconnections were found to promote twin propagation, the dislocations incorporated inside the twin are of considerable importance to hardening and damage initiation as they more significantly obstruct slip dislocations accommodating plasticity of the twins. In this work, the dislocation transmutation event and its effect on hardening is captured using a dislocation density based hardening model contained in a visco-plastic self-consistent mean-field model. This is done by allowing the twins to increase their dislocation densities, not only by virtue of slip inside the twin, but also through dislocations that transmute from the parents as the twin volume fraction increases. A correspondence matrix rule is used to determine the type of converted dislocations while tracking and parameterizing their evolution. This hypothesis provides a modeling framework for capturing slip-twin interactions. The model is used to simulate the mechanical response of pure Mg and provides a more physically based approach for modeling stress-strain behavior.

## 1. Introduction

The utilization of hexagonal-close-packed (HCP) magnesium (Mg) and magnesium based alloys is of interest to industry because of their low density and high specific strength. Their low crystal symmetry leads to mechanically anisotropic behavior, especially in highly textured polycrystals, in which significant nucleation and growth of {10$\bar{1}$2} tensile twins may occur. {10$\bar{1}$1} compression twinning also occurs in Mg when the 〈c〉-axis is compressed or contracted, and has been correlated with a high propensity of damage when formed inside {10$\bar{1}$2} tensile twins or after significant pyramidal 〈c+a〉 slip has occurred [1,2]. Several design approaches have been developed in an effort to produce Mg alloys with higher ductility, mainly relying on the idea that twinning should be reduced [3]. Additions of rare earth elements proved to be useful for improving formability at low temperature, primarily by means of texture weakening. However, energy absorption levels remained shockingly low under high rate regimes relevant to car crash deformation [4,5]. In order to expand the range of industrial applications of Mg alloys the way twinning affects hardening must be better understood and predicted.

Studies of single crystal magnesium revealed that slip along the basal and prismatic planes are often active during deformation, both possessing a relatively low CRSS when compared with other deformation modes [6,7,8]. However, in order to accommodate deformation along the 〈c〉-axis, pyramidal-〈c+a〉 slip and tensile {10$\bar{1}$2} twinning modes are often activated as well, leading to heavily anisotropic behavior, both observed experimentally and predicted in polycrystal modeling [9,10,11,12,13]. In some cases, highly profuse twinning in which 80% of the parent grain volume is overtaken by twin volume fraction can occur. In other cases, scarcely no twinning at all may take place [6,8]. The reason for this anisotropic behavior is well understood, and is rooted in the polarity of twinning combined with its marked low CRSS compared to any other deformation mode able to accommodate 〈c〉-axis strain. As {10$\bar{1}$2} twinning cannot facilitate 〈c〉-axis compression, the crystal or grain is very strong when compressed along the 〈c〉-axis by virtue of the domination of pyramidal-〈c+a〉 slip. However, compression perpendicular to 〈c〉 forces 〈c〉-axis extension. In this case the crystal is initially weak when compared to compression along along the 〈c〉-axis, but quickly hardens as the reorientation by twinning rotates the parent lattice, forcing it back into 〈c〉-axis compression. Similar anisotropy occurs under tension.

As highlighted in the work of Oppedal et al. [14], if the anisotropic behavior of Mg is informed exclusively by the process described above, then the saturation stress should, in principal, be roughly the same for both compression paths. But this is not the case, and consequently, additional hardening mechanisms are needed to explain this discrepancy. Previously reported crystal plasticity simulation work generally introduced Hall–Petch effects in order to obtain acceptable stress-strain simulation results [15,16,17,18]. If the differences in saturation stress associated with the incidence of twinning in Mg were motivated by Hall–Petch effects, then it is logical to expect the observation of dislocation pile-up at twin boundaries. However, pre-strained Mg polycrystals at various levels of strain showed that {10$\bar{1}$2} twins readily pass through any dislocation substructure that they encounter during their propagation, suggesting that twin boundaries are able to either absorb or incorporate those dislocations [2].

Oppedal et al. [14] go on to demonstrate that, rather than being caused by Hall–Petch effects, this anisotropy could be the result of increased dislocation density inside the twin grains. Implementing a modified version of the dislocation density based hardening model of Beyerlein and Tomé [19] in the visco-plastic self-consistent (VPSC) code (VPSC-7d from Los Alamos National Laboratory) the compression of rolled Mg with a highly basal texture along multiple compression loading paths were simulated. By setting the contributions of Hall–Petch mechanisms to zero, Oppedal et al. [14] showed that increasing the amount of dislocation density stored inside twin volumes by a Twin Storage Factor (TSF) recreated the characteristic hardening behavior across multiple load paths. This approach will be referred to as the TSF method in the following. This demonstrated that the assumptions about how slip dislocations interact with twin grain boundaries may require additional investigation in order to be completely understood. More specifically, it was shown that increasing the dislocation density of twin grains could reproduce hardening reactions previously thought to have been caused solely by Hall–Petch effects. However, this empirical approach was limited in that the specific physical process by which dislocation density was increased inside of twin grains was not modeled.

The findings of Oppedal et al. [14] were bolstered by the more recent studies of slip-twin interactions in the works of El Kadiri et al. [20], Barrett et al. [21], and Wang et al. [22], in which the behavior of basal dislocations encountering {10$\bar{1}$2} twin boundaries were investigated by means of molecular dynamic simulations and transmission electron microscopy. These studies showed that such dislocations readily disassociate into twinning disconnections bolstering twin propagation, interfacial disclination dipoles (facets), and other dislocations that transmute into the interior of the twin volume, called transmuted dislocations. Though the complete dislocation reaction at the twin interface was only recently identified by means of atomistic simulations and advanced interfacial defect theory, the part of the dissociation leading to dislocation transmutation has been hypothesized as early as the nineteen sixties by Bilby and Saxl and was later known as the Basinski mechanism [23,24,25]. Similar to the Basinski mechanism [25], the capacity of dislocations to transmute from parent grains across twin boundaries represents a potential vehicle by which the dislocation density of twin grains might be increased in excess of statistically and geometrically necessary dislocations typically formed to accommodate slip within the twin. Transmutation could then account for the increased dislocation density enforced by the twin storage factor of Oppedal et al. [14]. While previous studies have conceded that a Basinski type transmutation effect may be occuring in twinning Mg, experimental evidence has been sparse thus far [2,15,16,18]. The work of El Kadiri and Oppedal [26] provides a crystal plasticity framework by which such transmutation effects might be modeled in VPSC simulations. They developed a transmutation matrix α where each element $\alpha_{ij}$ represents the proportion of dislocation density that is transmuted to the *i*th slip mode inside the twin volume from the *j*th slip mode in the parent volume.

In order to investigate the potential role of dislocation transmutation effects in the hardening of Mg, the work presented herein presents a modified version of the crystal plasticity based dislocation transmutation theory of El Kadiri and Oppedal [26], further adapted to capture the system-wise nature slip dislocation interaction with twin boundaries and allow for the inclusion of both transmutation and dissociation behavior of these dislocations. In this way, a physically based model for the complex interactions of slip and twinning in polycrystal Mg is developed. This model is implemented in the LANL code VPSC-7d, and is calibrated against data from rolled Mg under multiple compression load paths. Finally, it is used to simulate the anisotropic hardening behavior of the material.

## 2. Model

The hardening model for this work is based on the approach of Beyerlein and Tomé [19] in which the hardening behavior of HCP polycrystals is simulated by the evolution of the critical resolved shear stress (CRSS) used in Hutchinson’s strain rate sensitive constitutive equation for slip. In this approach, the CRSS for slip for a given slip system *s* as part of the slip mode, *i*, is defined as a function of the dislocation density on that system. The CRSS of each system is given by the additive decomposition

(1) $$\tau_{c}^{s}\left(\rho^{i}\right)=\tau_{0}^{i}+\tau_{forest}^{i}\left(\rho^{i}\right)+\tau_{deb}^{i}\left(\rho^{i}\right)+\tau_{HP}^{s},\;\forall s\in i.$$

Here, $\tau_{0}^{i}$, $\tau_{forest}^{i}$, $\tau_{deb}^{i}$, and $\tau_{HP}^{s}$ are the initial critical stress values, hardening from forest dislocations, hardening from debris formation, and hardening from Hall–Petch effects, respectively. They can be defined as

(2a) $$\tau_{forest}^{i}\left(\rho^{i}\right)=b^{i}\chi \mu \sqrt{\rho^{i}},$$

(2b) $$\tau_{deb}^{i}\left(\rho^{i}\right)=k_{deb}\mu b^{i}\sqrt{\rho_{deb}}\log\left(\frac{1}{b^{i}\sqrt{\rho_{deb}}}\right),\;\mathrm{and}$$

(2c) $$\tau_{HP}^{s}=\left\{\begin{array}{c} \mu {\mathrm{HP}}^{i}\sqrt{\frac{b^{i}}{d_{g}}}\;\mathrm{for}\;\mathrm{grains}\;\mathrm{without}\;\mathrm{twins},\\ \mu {\mathrm{HP}}^{ij}\sqrt{\frac{b^{i}}{d_{mfp}^{s,PTS}}}\;\mathrm{for}\;\mathrm{grains}\;\mathrm{with}\;\mathrm{twins}. \end{array}\right.$$

In Equation (2a), $b^{i}$ and $\rho^{i}$ are the Burgers vector and dislocation density on the *i*th slip mode. χ and μ are the dislocation interaction coefficient and shear modulus, respectively. Equation (2b) also contains the debris formation parameter $k_{deb}$ and debris density $\rho_{deb}$. The Hall–Petch contributions in Equation (2c) depend on the presence of twins. In grains where there is no active twinning, these contributions are the same for all s∈i, with a bulk interaction term $HP^{i}$ and grain size $d_{g}$. For grains containing twins, the values of $\tau_{HP}^{s}$ cannot be assumed to be the same for all s∈i. In this case, the interaction term $HP^{ij}$ describes the interaction between twin mode *j*, which contains the predominate twin system, and slip mode *i*. Here, $d_{mfp}^{s,PTS}$ is the mean free path between twin lamella for dislocations on slip system *s* as described in Proust et al. [17].

In the simulations presented in this work, the forest and debris contributions remain unchanged from the forms presented in Beyerlein and Tomé [19]. However, the constitutive model of Beyerlein and Tomé [19] is based on the Composite Grain (CG) model of Proust et al. [17] and treats twin boundaries as full-stop barriers to dislocation glide. As twin boundaries are not considered to act as barriers in this work by hypothesis, the Hall–Petch contributions to CRSS are treated as being identically zero, hence

(3) $$\tau_{HP}^{s}=0,\;\forall s\in i.$$

In the model of Beyerlein and Tomé [19], the change in dislocation density for a given slip mode α with change in shear on the same system is calculated by a Mecking-Kocks [27] type equation and adopted in this work to calculate the change in dislocation density with shear as

(4) $$\frac{\partial \rho^{i}}{\partial \gamma^{i}}=k_{1}^{i}\sqrt{\rho^{i}}-k_{2}^{i}\left(\dot{\epsilon },T\right)\rho^{i}.$$

Equation (4) introduces the phenomenological generation and rate and temperature dependent parameters $k_{1}^{i}$ and $k_{2}^{i}$. In Beyerlein and Tomé [19], this leads to a relationship between these two parameters,

(5) $$\frac{k_{2}^{i}\left(\dot{\epsilon },T\right)}{k_{1}^{i}}=\frac{\chi b^{i}}{g^{i}}\left(1-\frac{kT}{D^{i}{\left(b^{i}\right)}^{3}}log\left(\frac{\dot{\epsilon }}{{\dot{\epsilon }}_{0}}\right)\right).$$

Above, $g^{i}$, $D^{i}$ are the normalized activation energy and drag stress, respectively, and $\dot{\epsilon }$ and ${\dot{\epsilon }}_{0}$ are the strain rate and reference strain rate.

In the present model, the dislocation densities were modified based on changes in twin volume fraction of the composite grain, which were added:

(6) $$\rho^{iTF}=\left\{\begin{array}{cc} \rho^{i}, & \forall s\in \mathrm{parent}\;\mathrm{volume}\;\mathrm{fraction}\\ \rho^{iTF}\left(V\right), & \forall s\in \mathrm{parent}\;\mathrm{volume}\;\mathrm{fraction}. \end{array}\right.$$

In this way, the hardening contributions from dislocation density to the CRSS of slip systems inside the twin volume fraction of each grain are differentiated from those systems inside the parent volume fraction.

The function $\rho^{iTF}\left(V\right)$ from the work of El Kadiri and Oppedal [26] defining the increase in dislocation density of the *i*th slip mode type inside the *j*th twin volume fraction is expressed as

(7) $$d\rho^{iTF}\left(V\right)=\frac{V^{T}\rho^{iT}+dV\sum_{j}\alpha_{ij}\rho^{jP}}{V^{T}+dV}.$$

In the present work, the matrix α includes an additional row in order to account for dislocation transmutation into sessile, higher order dislocations and Equation (7) is modified in order to account for the dissociation of parent dislocations at twin grain boundaries. A normalized dissociation parameter η is introduced, representing the proportion of dislocation density that fails to transmute from the *j*th slip system in the parent volume to the twin volume and does not contribute to dislocation density in the twin. This leads to the final, modified constitutive law

(8) $$d\rho^{iTF}\left(V\right)=\frac{V^{T}\rho^{iT}+dV\sum_{j}\left(1-\eta_{j}\right)\alpha_{ij}\rho^{jP}}{V^{T}+dV}.$$

The model of Beyerlein and Tomé [19] also contains constitutive laws for twin activation, propagation, and interaction. These remained unchanged in the current work and are summarized in Appendix A.

## 3. Implementation and Calibration

The above described changes to the dislocation density model were implemented in the LANL code VPSC-7b by making changes directly to the subroutines UPDATE_CRSS_CG_disl and DATA_CRYS and were compiled using the GFortran GNU compiler. The model was calibrated using a three stage process. First, the values for α were assigned based on the correspondence method introduced by Bilby and Crocker [23]. After this, values for the elements $\eta_{j}$ were assigned, and finally, the slip and twinning parameters respectively associated with dislocation generation, twin nucleation, and twin propagation were adopted from Oppedal et al. [14].

### 3.1. Correspondence Method for Transmutation for the Construction of the α matrix

A generalized correspondence method for mapping a vector on a slip plane in a parent grain on to a corresponding slip plane inside a twin grain was first presented in the work of Bilby and Crocker [23]. This method was applied to twin modes in FCC and HCP crystals in the works of Niewczas [28,29]. Summarized in Appendix B, the precise mathematical expression of the general theory of the correspondence method shown in Niewczas [29] in the case of Mg was adapted for the purpose of assigning values to the transmutation matrix α.

The onto mapping of slip systems in the parent volume to corresponding slip systems in the twin volume implemented by Niewczas [29] provides explicit calculations for transmuting the plane normal and directional vectors for each slip system in a parent grain to its corresponding slip system inside a twin. Rather than using the method to calculate values of α at each deformation step, the transmutation matrix for {10$\bar{1}$2} twins was constructed in pre-processing. {10$\bar{1}$1} twins were not assumed to contribute to transmutation in this work as the onset of compression twinning in Mg is taking place at higher strains, and very closely associated with the nucleation of damage in the material, leading quickly to brittle failure. As such, the contributions of compression twins to dislocation transmutation were assumed to be negligible. Strictly for the purposes of the element wise construction of α, the following assumptions were made:

1. The entirety of the dislocation density from a slip system in the volume fraction of the parent grain overtaken by a twin mode is considered to transmute to its corresponding slip system inside the twin grain. This pairing of slip systems is defined by the mappings defined by the correspondence method used by Niewczas [29] for HCP materials.
2. The dislocation density of slip mode in the volume fraction of the parent grain overtaken by a twin volume is considered to be evenly distributed across the systems of that mode.
3. In this simulation, the dislocation density of any parent slip system corresponding to a slip system inside the twin grain that was not part of the prismatic, basal, or 2nd order pyramidal 〈c+a〉 slip modes was assumed to contribute to debris formation inside the twin as part of $\rho_{deb}$.

Working from these assumptions, the value of each element $\alpha_{ij}$ can be calculated as the proportion of systems from the *j*th slip mode in the parent volume fraction which contribute to dislocation density in the *i*th slip mode in the twinned volume. This can be expressed as

(9) $$\alpha_{ij}=\frac{n_{j}^{C}}{n_{j}^{tot}},$$

where $n_{j}^{C}$ is the number of slip systems in the *j*th slip mode of parent volume mapped onto slip systems in the *i*th slip mode inside the twin volume and $n_{j}^{tot}$ is the total number of slip systems in the *j*th slip mode of the parent volume. The values of $\alpha_{ij}$ for Mg used in this work are summarized in Appendix C.

### 3.2. Parameters for Dissociation

The molecular dynamics simulations in the work of El Kadiri et al. [20] suggest that the type of dislocation and its orientation relative to the advancing twin boundary play a significant role in determining whether a given dislocation will transmute across the boundary or dissociate upon contact. In the simulations presented in this work, screw type basal dislocations were allowed to transmute perfectly across tensile twin boundaries. Edge type dislocations would either transmute across these twin boundaries or dissociate into twinning disconnections according to their orientation relative to the twin boundary, with positively oriented dislocations transmuting across the boundary and negatively oriented ones dissociating.

The simulations presented in this work were conducted at relatively low strain regimes, with ϵ≤0.25 for all simulations. As such, the following assumptions were made:

1. Dislocations in the prismatic, basal, and 2nd order pyramidal 〈c+a〉 modes are assumed to transmute.
2. The incidence of screw type dislocations at low strain regimes is quite low. As such, for the purposes of this work, it was assumed that the contributions to dislocation density inside the twin volume fractions made by these types of dislocations were negligible.
3. Relative to twin boundaries, it was assumed that dislocations with positive and negative Burgers vector occur in equal measure.

The proportion of dislocation density dissociated at twin boundaries rather than transmuted is set by the η parameter at

(10) $$\eta_{prismatic}=\eta_{basal}=\eta_{pyramidal}=\eta_{higherorder}=0.5,$$

and summarized again in Appendix C.

#### Parameters for Dislocation Generation and Twin Nucleation and Propagation

Simulations from the work of Oppedal et al. [14] were taken as a baseline for the presented work, and parameters for the constitutive laws governing dislocation generation, drag stress, normalized activation energy, critical stress values, and deactivated Hall–Petch effects were taken from this work. Transmutation effects were used in place of twin storage factor, reducing the latter value to zero. The values of these parameters are summarized in Appendix A.

### 3.3. Simulation

**Figure 1:**
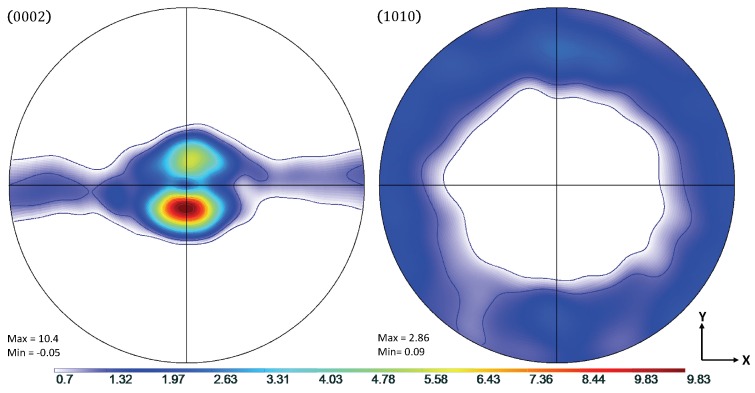
Pole figures showing the initial rolled texture of the Mg specimen.

Experimental data from the work of Oppedal et al. [14] was utilized to establish a control data set, where possible. In the case where such data were not available, comparisons were made to simulated results from the same work. In this work, rolled, pure magnesium with a highly basal texture in the normal direction was subjected to uniaxial compression along the normal and transverse directions, hereafter referred to as through thickness compression (TTC) and in-plane compression (IPC), respectively. This was done in order to capture the anisotropic mechanical behavior of textured magnesium, and, in particular, to motivate both scant and profuse twin volume growth. The chemical composition of the pure magnesium used to generate the experimental data in Oppedal et al. [14] is summarized in Table 1. The initial texture data was obtained via neutron diffraction at LANL and pole figures generated from this data are shown in Figure 1.

**Figure 2:**
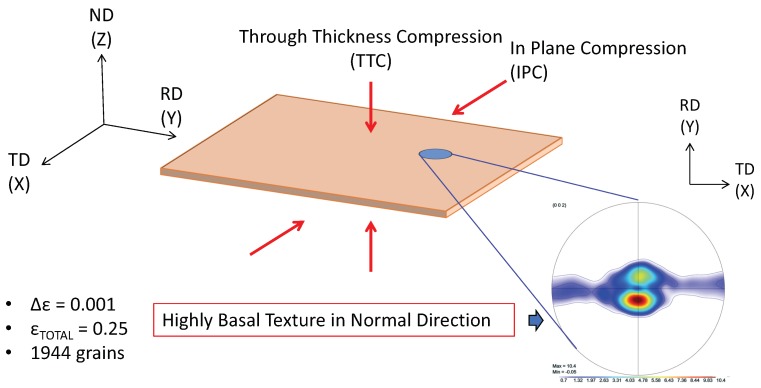
Simulated load paths and initial texture for simulations of rolled pure magnesium.

Shown in Figure 2, the load paths for the simulations presented in this work were performed along both TTC and IPC directions on polycrystals consisting of 1944 grains with a highly basal texture in order to recreate the conditions under which the literature data were collected. The loading was conducted at room temperature conditions, with a strain increment of Δϵ=0.001, up to a total compressive true strain of ϵ=0.25. The initial texture of the compressed Mg specimen is shown in Figure 1.

## 4. Results

### 4.1. TTC Load Path

**Figure 3:**
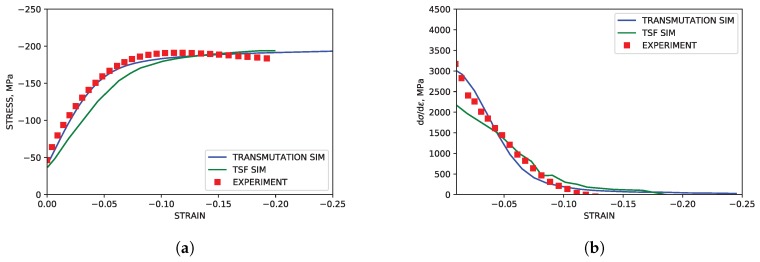
Simulated and experimental mechanical response under TTC compression. (**a**) Stress-strain. (**b**) Hardening rate vs. strain.

**Figure 4:**
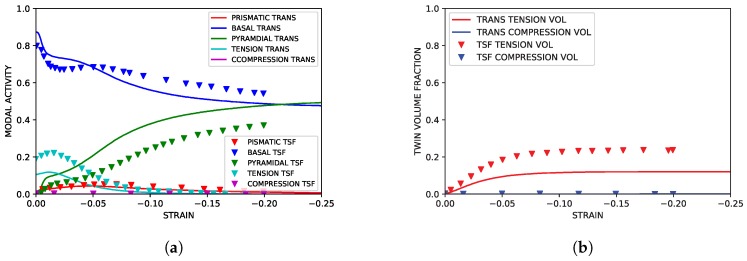
Simulated slip and twinning activity under TTC compression compared to simulated results from Oppedal et al. [14]. (**a**) Simulated modal contributions vs. strain. (**b**) Simulated twin volume growth vs. strain.

As highlighted in Figure 3, the TTC simulations reproduced both the texture and hardening curves from the experimental literature data with good accuracy. Although the strain softening observed in the experimental data at approximately ϵ=0.1 was not reproduced in the simulation, capturing such behavior lies beyond the scope of the current work. The simulated modal activity, being defined as the proportional contribution to strain from each mode at each deformation increment, and evolution of twin volume fraction shown in Figure 4, both followed trends seen in simulations utilizing twin storage factor effects. Good agreement between the two simulation approaches was observed, however, tension twin volume was somewhat under predicted relative to simulations utilizing the twin storage factor approach.

**Figure 5:**
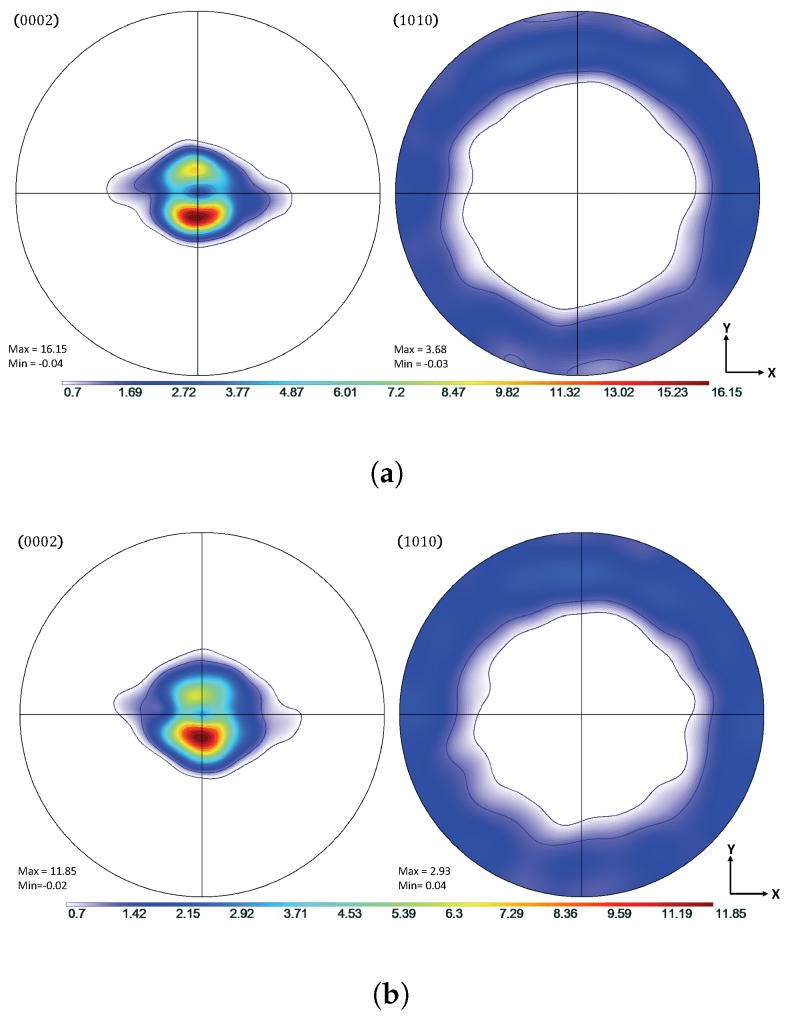
Comparison of simulated and experimental textures under TTC load conditions at ϵ = 0.09. (**a**) Simulated {0002} and {10$\bar{1}$0} pole figures. (**b**) Experimental {0002} and {10$\bar{1}$0} pole figures.

From the literature, the texture in TTC simulations was not expected to change much over the course of loading. This was evident in Figure 5 showing results of the simulations utilizing the transmutation scheme, with the basal texture remaining consistent throughout the compression loading.

### 4.2. IPC Load Path

**Figure 6:**
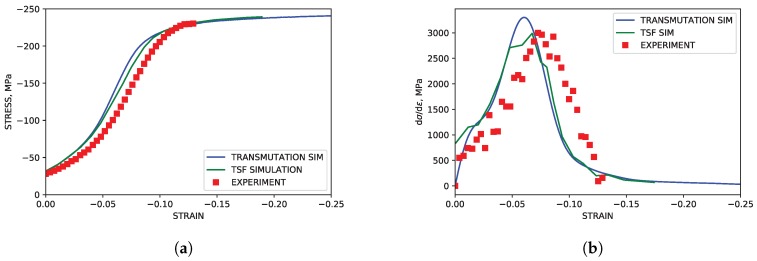
Simulated and experimental mechanical response under IPC compression. (**a**) Stress-strain. (**b**) Hardening rate vs. strain.

**Figure 7:**
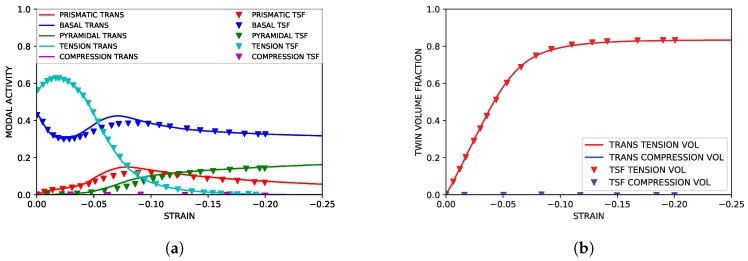
Simulated parent slip and primary twinning activity under IPC compression compared to simulated results from Oppedal et al. [14]. (**a**) Simulated modal contributions vs. strain. (**b**) Simulated twin volume growth vs. strain.

**Figure 8:**
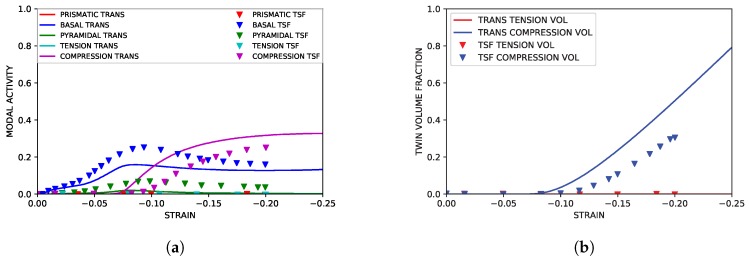
Simulated twin slip and secondary twinning activity under IPC compression compared to simulated results from Oppedal et al. [14]. (**a**) Simulated modal contributions to strain vs. strain. (**b**) Simulated twin volume growth vs. strain.

The sigmoidal stress curve associated with a high incidence of twinning was successfully captured in the IPC simulations. The associated simulated hardening rate was reasonably similar to experimental data from the literature. These results are shown in Figure 6. Shown in Figure 7 and Figure 8, modal contributions to strain were nearly identical to simulation results from previous simulation literature as well. The growth of secondary compression twin volume fraction did exceed that seen in simulations utilizing the twin storage factor approach, but it is assumed that this can be neglected as the onset of compression twinning is, at higher strains, associated with void nucleation along compression twin grain boundaries leading to brittle failure. This fact was reinforced by the failure of the experimental control specimen coinciding with the formation of significant (>10%) volume fraction of secondary compressive twins within the primary twin volume.

**Figure 9:**
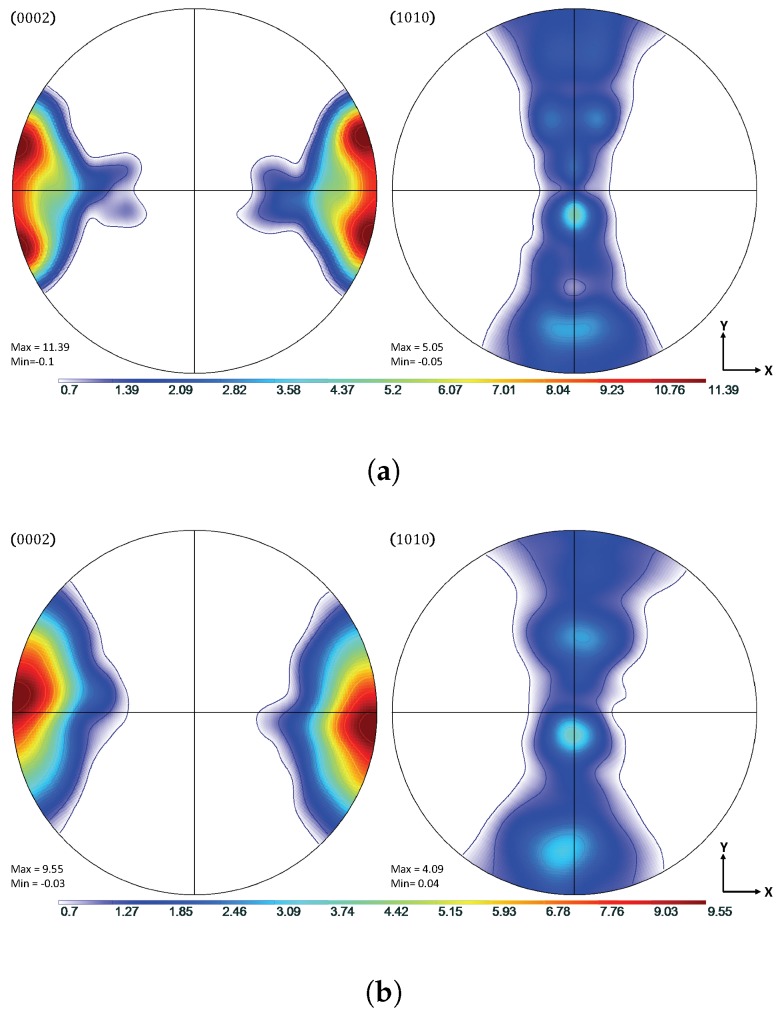
Texture comparison of simulated and experimental textures under IPC load conditions at ϵ = 0.12. (**a**) Simulated {0002} and {10$\bar{1}$0} pole figures. (**b**) Experimental {0002} and {10$\bar{1}$0} pole figures.

In Figure 9, the textural evolution is shown to be consistent with experimental data from the literature, with high concentrations of orientations in the neighborhood of 90° from the normal specimen axis. This type of reorientation is consistent with the high degree of tensile twinning.

## 5. Discussion

As the evolution of yield stress and twin volume with strain of pure magnesium is heavily anisotropic, it becomes necessary to consider a measure for “goodness of fit” of simulated data along multiple load paths when evaluating the degree to which the transmutation model and the method for calibrating its parameters are validated. This measure must also be extended to simulated data obtained from simulations utilizing the TSF model in order to make truly meaningful conclusions regarding connections between the mechanical behavior resulting from artificially increased dislocation density in twin grains and the increased dislocation density resulting from transmutation effects. To this end the normalized mean squared error (NMSE) was calculated across the simulated strain sub-domains for which experimental data was available (ϵ=0 to 0.2 for TTC and ϵ=0 to 0.125 for IPC). Summarized in Table 2, these values were calculated for both load paths and for both modeling approaches.

For simulations of the TTC load path, the normalized mean squared error of the transmutation model is approximately 10% lower than that of the TSF model. In the IPC load path simulations, the normalized mean squared error is roughly 5% higher. Taking both of these comparisons into consideration, it can be concluded that while the transmutation model does suffer from some loss of accuracy in IPC simulations compared to the TSF approach, this loss is marginal at worst and more than offset by gains in accuracy in TTC simulations. This conclusion is further reinforced when one considers that the TSF model is a more empirical approach and, therefore, more limited than transmutation model by the availability of sound experimental data to insure its predictive capabilities.

### η Sensitivity

Although stress-strain curves, texture evolution, and modal activities were consistent both with previous experimental and simulation work, further validation of the modeling approach is necessary to conclude that predicted results are indeed the product of increased dislocation density inside the twinned volume fraction of the simulated polycrystal.

**Figure 10:**
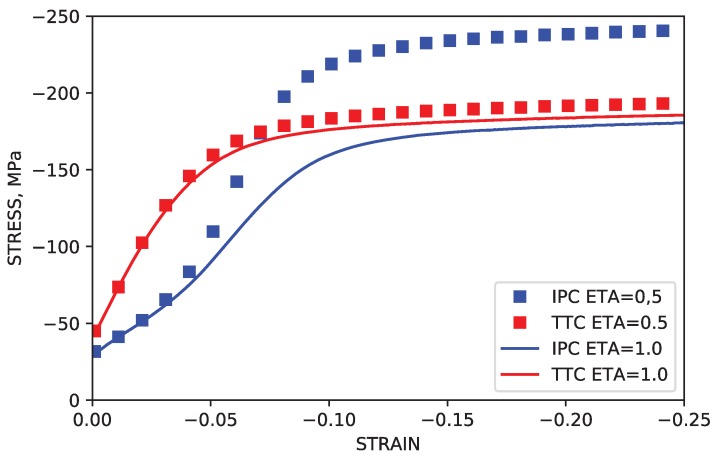
Simulated stress-strain curves with a dissociation parameter of η = 0.5 and dissociation parameter η = 1.0. These values correspond to a state of active dislocation transmutation and a state of no transmutation, respectively.

In experiments and in simulations using both the TSF method and the method from the current work, the saturation stress observed in the TTC and IPC load paths differs significantly by approximately 60 MPa. In order to confirm that the “gulf” observed between the saturation stress levels of TTC and IPC simulations was the result of increased hardness due to higher dislocation density brought on by dislocation transmutation, additional simulations in which transmutation effects were deactivated were performed for both load paths. This corresponds to simulations in which all of the dislocations dissociate upon reaching the twin boundaries, leading to dissociation parameter values of η=1.0. In these cases, the saturation stress levels for both TTC and IPC simulations were shown to be nearly equal, at roughly $\sigma_{saturation}=180$ MPa. These results are summarized in Figure 10.

**Figure 11:**
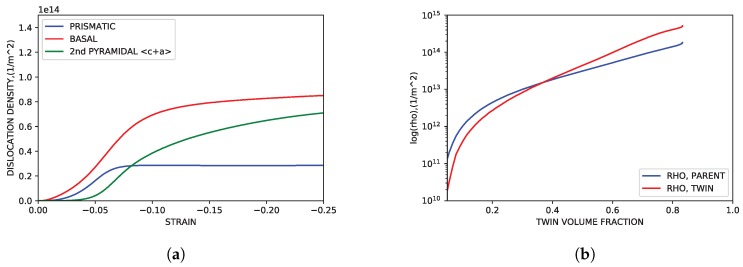
Simulated dislocation density evolution. (**a**) Modal dislocation density vs. strain. (**b**) Dislocation density for parent and primary twin volume fractions vs. primary twin volume fraction.

Figure 11a shows the evolution of the dislocation density of each slip as a function of IPC compression. Dislocation activity correlates well with the modal activity trends shown in Figure 7a, showing increasing dislocation density across strain sub-domains in modes with more modal activity. As shown in Figure 11b, the dislocation density evolution in IPC simulations is consistent with the TSF parameters of Oppedal et al. [14]. At the point in which twin volume fraction was fully realized (roughly 80% total volume fraction) the dislocation density of the twin volume fraction lies in the range of twice that of the the dislocation density of the parent volume fraction.

**Figure 12:**
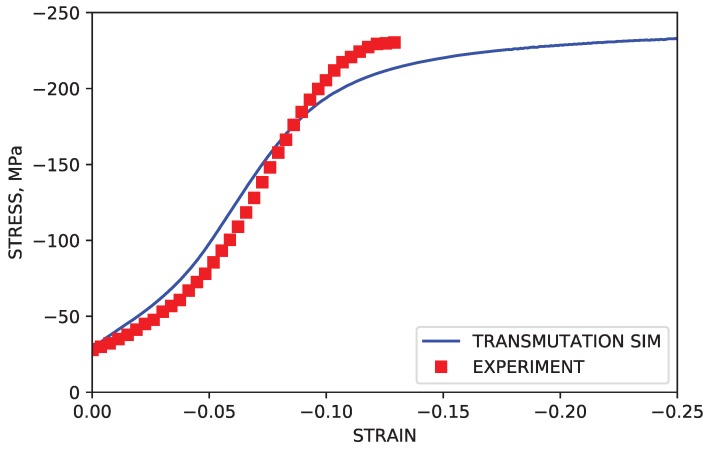
Simulated IPC stress curve with transmutation active in the basal mode only.

The assumption that dislocations exist on the prismatic 〈a〉 and 2nd order pyramidal 〈c+a〉 was also put to the test. IPC compression was simulated under the assumption that only basal dislocations would be allowed to transmute across twin grain boundaries. This corresponds to dissociation input parameters of $\eta_{basal}=0.5$ and $\eta_{prismatic}=\eta_{2^{nd}\;order\;pyramidal}=1.0$. Shown in Figure 12, the stress levels after the onset of twinning were noticeably under predicted. While stress levels for simulations with only basal dislocations actively transmuting were closer to approximating experimental stress levels than simulations in which no transmutation was activated, the relative similarities can be accounted for by acknowledging that deformation by basal slip, and therefore basal type dislocation density, is significantly higher than both prismatic 〈a〉 and 2nd order pyramidal 〈c+a〉 in general. This is supported by observations of simulated dislocation density by mode, summarized in Figure 11a.

## 6. Conclusions

In summary, methods for simulating the effects of twin transmutation and dissociation at twin grain boundaries were implemented in a visco-plastic self-consistent framework in place of more conventional approaches that utilize Hall–Petch effects, and used to simulate uniaxial compression along multiple load paths in order to capture the behavior of magnesium under conditions which twin volume growth was both profuse and sparse. The results of these simulations were compared to both experimental data and simulated data from previous approaches. The results permit highlighting the following points:

1. In place of Twin Storage Factor and Hall–Petch effects, a model for dislocation and twin boundary interactions was implemented. These methods and parameter selections were used to simulate the behavior of pure, basal textured, rolled magnesium subjected to uniaxial compression along (TTC) and perpendicular to (IPC) the dominant 〈c〉-axis of the texture. The simulation results for stress, hardening, and texture development were consistent with observed experimental results. Modal activity and twin volume growth were largely similar to simulation work performed using the TSF approach.
2. The large difference between the saturation stress of the simulated IPC and TTC load paths was confirmed to be the result of transmutation effects included in the model by disabling the contributions of transmuted dislocations to the dislocation density of twin volume fractions.
3. It can be stated that Hall–Petch effects cannot be assumed to be the sole or even primary source of twinning induced anisotropy in the mechanical behavior of pure magnesium.

## Figures and Tables

**Table 1:** Composition of pure magnesium in ppm.

Al	Ca	Mn	Zr	Zn	Sn	Si	Pb	Mg
30	10	40	10	130	10	40	10	Balance

**Table 2:** Normalized mean squared error for transmutation and TSF modeling approach.

	NMSE
Transmutation (TTC)	5.7613%
TSF (TTC)	16.0268%
Transmutation (IPC)	21.0105%
TSF (IPC)	16.7439%

## Abbreviations

The following abbreviations are used in this manuscript:

VPSC	Visco-Plastic Self-Consistent
HCP	Hexagonal Close Packed
Mg	Magnesium
CRSS	Critical Resolved Shear Stress
HP	Hall–Petch
LANL	Los Alamos National Laboratory
TSF	Twin Storage Factor
MD	Molecular Dynamics
CG	Composite Grain
TTC	Through Thickness Compression
IPC	In-Plane Compression

## Appendix A. Parameters for Dislocation Density Based Hardening

Constitutive equations for twin hardening evolution in Beyerlein and Tomé [19]:

(A1) $$\tau_{c}^{t}=\tau_{0}^{k}+\tau_{HP}^{t}+\tau_{slip}^{k}$$

(A2) $$\tau_{HP}^{t}=\left\{\begin{array}{c} \frac{HP^{k}}{\sqrt{d_{g}}}\;\mathrm{no}\;\mathrm{twinning}\;\mathrm{or}\;\mathrm{when}\;t=\;\mathrm{predominate}\;\mathrm{twin}\;\mathrm{system},\\ \frac{HP^{kk^{\prime }}}{\sqrt{d_{mfp}^{s}}},\;\mathrm{when}\;t\neq \;\mathrm{predominate}\;\mathrm{twin}\;\mathrm{system}. \end{array}\right.$$

(A3) $$\tau_{slip}^{k}=\mu \sum_{i}C^{ik}b^{k}b^{i}\rho^{i}$$

Hardening and dislocation generation parameters adopted from Oppedal et al. [14]:

**Table A1:** Parameters for hardening evolution of slip.

	Prismatic	Basal	Pyramidal〈c+a〉
$k_{1}$ ${}^{1}{/}_{m}$	2.0 × $10^{9}$	0.25 × $10^{9}$	2.0 × $10^{9}$
$\dot{\epsilon }$ $s^{-1}$	1.0 × $10^{7}$	1.0 × $10^{7}$	1.0 × $10^{7}$
$g^{\alpha }$	0.0035	0.0035	0.003
$D_{0}^{\alpha }$ MPa	3.4 × $10^{3}$	10.0 × $10^{3}$	0.08 × $10^{3}$
$\tau_{0}^{\alpha }$ MPa	30	11	50
χ	0.9	0.9	0.9
$HP^{\alpha }$	0	0	0
$HP^{\alpha \beta }$	0	0	0

**Table A2:** Parameters for hardening evolution of twinning.

	Tensile Twin	Compression Twin
$\tau_{0}^{\beta }$ MPa	$\tau_{crit}=15,\;\tau_{prop}=10$	$\tau_{crit}=185,\;\tau_{prop}=185$
$HP^{\beta }$	0	0
$HP_{TW}^{\beta \beta }$	0	0
$C^{\beta 1}$	0	0
$C^{\beta 2}$	0	0
$C^{\beta 3}$	0	0

## Appendix B. Correspondence Method; Summary of Equations

Presented here is a summary of the general Correspondence Method equations from Niewczas [28], Niewczas [29] and Bilby and Crocker [23]. In this, a vector $u_{M}$ on in the parent lattice is mapped onto the vector $v_{T}$ in the twin vector using the matrices S, X, and U. These matrices are constructed from elements of m and n describing the twinning direction and normal to the twin plane.

(A4) $$S=\left[\begin{array}{ccc} 1+sm_{1}n_{1} & sm_{1}n_{2} & sm_{1}n_{3}\\ sm_{2}n_{1} & 1+sm_{2}n_{2} & sm_{2}n_{3}\\ sm_{3}n_{1} & sm_{3}n_{2} & 1+sm_{3}n_{3} \end{array}\right]$$

(A5) $$R=\left[\begin{array}{ccc} -1 & 0 & 0\\ 0 & -1 & 0\\ 0 & 0 & 1 \end{array}\right]$$

(A6) $$X=\left[\begin{array}{ccc} m_{1} & m_{2} & m_{3}\\ m_{3}n_{2}-m_{2}n_{3} & m_{1}n_{3}-m_{3}n_{1} & m_{2}n_{1}-m_{1}n_{2}\\ n_{1} & n_{2} & n_{3} \end{array}\right]$$

(A7) $$v_{M}=Su_{M}$$

(A8) $$U=X^{-1}RX$$

(A9) $$v_{T}=USu_{M}.$$

## Appendix C. Parameters for Transmutation and Dissociation: Alpha Matrix Composition

Final values of transmutation matrix α used in simulations of Mg, with prismatic, basal, 2nd order pyramidal 〈c+a〉 modes and debris corresponding to indices 1,2,3, and 4, respectively:

(A10) $$\alpha =\left[\begin{array}{ccc} 0.0 & 0.333\bar{3} & 0.333\bar{3}\\ 0.333\bar{3} & 0.0 & 0.0\\ 0.666\bar{6} & 0.0 & 0.0\\ 0.0 & 0.666\bar{6} & 0.666\bar{6} \end{array}\right]$$
